# Test of IL28B Polymorphisms in Chronic Hepatitis C Patients Treated with PegIFN and Ribavirin Depends on HCV Genotypes: Results from a Meta-Analysis

**DOI:** 10.1371/journal.pone.0045698

**Published:** 2012-09-21

**Authors:** Zhifang Jia, Yanhua Ding, Suyan Tian, Junqi Niu, Jing Jiang

**Affiliations:** 1 Division of Clinical Epidemiology, First Hospital of Jilin University, Changchun, China; 2 Department of Phase I Clinical Trials of Medicine, First Hospital of Jilin University, Changchun, China; 3 Department of Hepatology, First Hospital of Jilin University, Changchun, China; University of North Carolina School of Medicine, United States of America

## Abstract

**Background:**

Many studies have been published on the association between single nucleotide polymorphisms (SNP) near the IL28B gene and response to the combined treatments of pegylated-interferon (PegIFN) and ribavirin (RBV) in chronic HCV-infected patients, but without identical conclusions. The aim of this study was to assess impact of the IL28B polymorphisms on the effect of HCV standard treatment using meta-analysis based method.

**Methods:**

Association studies between polymorphisms of rs12979860 or rs8099917 and response to PegIFN/RBV treatment in chronic HCV patients were retrieved from PubMed. Data of qualified studies on sustained virological response (SVR) in different genotypes were extracted and analyzed using meta-analysis method in Stata 10 software.

**Results:**

Thirty-four papers, containing 46 independent studies, were included in the analysis. In the HCV G1/4 patients without treatment history, individuals carrying rs12979860 CC genotype were more likely to achieve SVR (OR 3.97, 95%CI 3.29–4.80) compared to those carrying CT/TT genotypes. Similar results were observed in the HCV G1/4 patients with unsuccessful or unknown treatment history (OR 3.76, 95%CI 2.67–5.28) or in the patients co-infected with human immunodeficiency virus (OR 5.20, 95%CI 3.04–8.90). However, associations could not be observed in HCV G2/3 patients. For rs8099917, similar results were obtained for genotype TT compared to genotypes TG/GG, indicating that TT genotype was significantly associated with better treatment response in patients infected with genotype 1 or 4 HCV, but not genotype 2 or 3 HCV.

**Conclusion:**

Polymorphisms of rs12979860 and rs8099917 near IL28B only associate with the treatment response to PegIFN/RBV in patients infected with HCV genotype 1 or 4 but not with genotype 2 or 3, irrespective of the previous treatment history or HIV co-infected status. Therefore, identification of IL28B genotypes is necessary only in patients infected with relatively difficult-to-treat genotype 1 or 4 HCV.

## Introduction

About 170 million people are affected with hepatitis C virus (HCV) throughout the world and 70% of them develop chronic infection which may progress to cirrhosis and hepatocellular carcinoma [1]. The current recommended treatment for chronic HCV infection is a combination of pegylated interferon (PegIFN α-2a or PegIFN α-2b) plus body-weighted ribavirin (RBV) for a duration of 24 weeks or 48 weeks depending on the HCV viral genotypes. However, only about 50% genotype 1 or 4 patients treated and 80% genotype 2 or 3 patients treated could respond completely and achieve sustained virological response (SVR) [2]. Moreover, side effects from the therapy such as influenza-like symptoms, psychiatric symptoms and hematological abnormalities, could result in the dose reduction or even the premature discontinuation of the treatment [3]. To avoid these potential adverse events in patients who do not benefit from the treatment and to reduce the cost of therapy, it is necessary to predict an individual's response before or at the early stage of the treatment. Several factors, viral or host, such as HCV genotypes, baseline viral load, liver fibrosis, and mutations of interferon sensitivity determining region (ISDR), have been reported to be linked to the treatment outcomes [3]–[5]. However, these factors still could not fully predict the therapy response.

In 2009, three studies which were published almost at the same time reported that single nucleotide polymorphisms (SNP) near IL28B gene region were associated with the treatment effect of pegylated-interferon and ribavirin (PegIFN/RBV) in HCV-infected patients using genome-wide association study (GWAS) method [4], [6], [7]. Thereafter, a number of studies were published on the association between SNPs near IL28B and the clearance of HCV with or without treatments in HCV infected subjects in different ethnicities and HCV genotypes [8]–[12]. The most studied two SNPs, rs12979860 and rs8099917, are located upstream to the IL28B gene [4]. The CC genotype of rs12979860 or TT of rs8099917 was considered to be associated with a better treatment response. However, results were not consistent from different studies. The aim of this study was to summarize the associations between SNPs (rs12979860 and rs8099917) near IL28B gene and outcome of the combination therapy of PegIFN plus RBV in chronic HCV infected patients from public data.

## Methods

### Study search and selection

Studies on the associations between SNPs of rs12979860 or rs8099917 and the treatment response in HCV infected patients were retrieved from PubMed using the following strategy: (“IL28B” OR “IL-28B” OR “interleukin 28B” OR “interleukin-28B” OR “interferon lambda 3” OR “IFN lambda 3” OR “rs12979860” OR “rs8099917”) AND (“Hepatitis C” OR “HCV”). No language or time restrictions were applied and database searching for the last time was November 20, 2011. Papers retrieved were reviewed by two reviewers (Jia and Ding) independently following the including and excluding criteria (File S1). References of retrieved publications were also screened manually to search potential articles fitted the criteria.

Research studies met the following inclusion criteria were included in the analysis. (1) chronic HCV-infected patients with detectable HCV RNA before treatment and received the combined therapy of peginterferon and ribavirin only; (2) the clear outcomes defined as sustained virological response (SVR), i.e. undetectable HCV RNA 24 weeks or 6 months after the cessation of treatment, or non-SVR, which was detectable HCV RNA at the end of follow-up or premature discontinuation of treatment because of the poor response; (3) relevant data of therapeutic effect stratified by HCV genotypes as different HCV genotypes respond differently to the therapy.

Studies were excluded if patients suffered from other complicated liver diseases such as hepatocellular carcinoma or underwent liver transplantation. If more than one articles reported overlapping subjects, the one with more detailed descriptions of baseline information and effect data was selected. When results were stratified by races or HCV genotypes in the article, each part was considered as an independent study. However, HCV genotypes were classified into two categories, G2/3 (genotype 2 or 3) and G1/4 (genotype 1, 4 and others), according to response to the combined treatment [2], [13]. When results of treatment per protocol (TPP) analysis and intention to treatment (ITT) analysis were both reported, only ITT data were extracted for the analysis.

The following information was extracted from each study using the assessment form for data collection (File S1): the first author, date of publication, area of the study, demographic data of subjects, HCV genotype, duration of therapy, and the effect data of each IL28B genotype.

### Data analysis

Meta-analysis was used to summarize associations across studies between IL28B genotypes and SVR of the combined treatment. Though studies performed in Asian populations reported a strong linkage disequilibrium between rs12979860 and rs8099917 [14]–[16], results from Caucasians showed a weaker linkage disequilibrium [4], [17], [18]. Therefore, we summarized effects of two SNPs separately. For each SNP, a recessive model was applied (for rs12979860, CC vs CT+TT; and for rs8099917, TT vs TG+GG). OR (odds ratio) of each study with its 95% CI (confidence interval) was calculated and displayed in a forest plot. Heterogeneity across studies was tested by Cochran's *Q* statistic and quantified by *I^2^*, a transformation of *Q* that estimates the percentage of variation in effect size that is due to heterogeneity. However, the pooled ORs were summarized using random effect model (DerSimonian and Laird method) even in absence of heterogeneity, given the diversity of the study design.

If patients fail in previous interferon-based treatment or are co-infected with human immunodeficiency virus (HIV), they respond differently to the therapy compared to patients with HCV mono-infection and treatment naïve [3]. Therefore, separate analyses were performed to reduce the potential heterogeneity. Furthermore, in treatment-naïve patients, summarized ORs stratified by races were calculated separately in G1/4 patients and G2/3 patients. Potential publication bias was assessed by Egger method and Peter method separately. All analyses were carried out in Stata software (version 10, Package sbe24_3 and sbe19_6) and a two-tailed *P* value less than 0.05 was considered as a statistical significance.

## Results

### Literature retrieved

**Figure 1:**
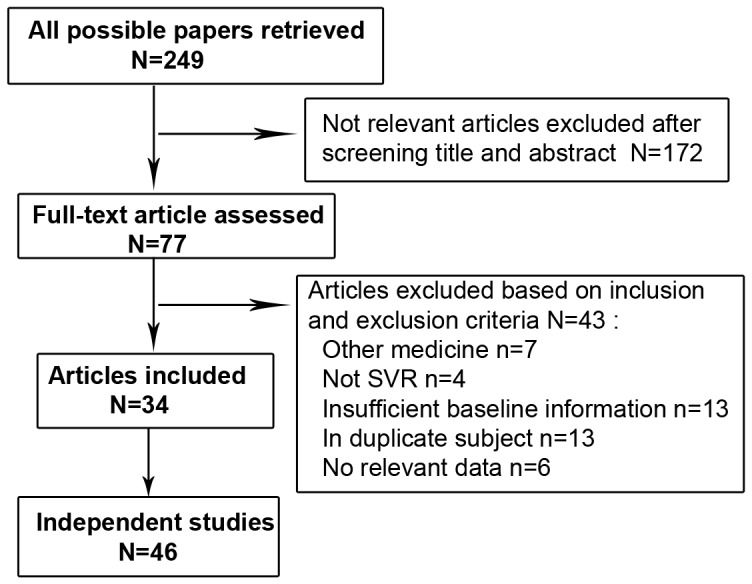
Flow chart summarizing the process of study selection.

The process of literature selection is showed in figure 1. Two hundred and forty-nine articles met the keyword selection and the full texts of 77 were gone through thoroughly after initially screening titles and abstracts. Forty three of 77 were excluded according to the inclusion and exclusion criteria. Table 1 shows the detailed information of 34 articles, including 46 independent studies [4], [8], [14]–[17], [19]–[46]. Since only one study was conducted in Hispanic patients [4], we incorporated it into the Caucasian group. Among those studies, 30 were on patients with HCV G1/4 and the summarized SVR rate was 45.3% (3600/7944). In contrast, the SVR rate in the rest 16 studies on patients infected with HCV G2/3 was much higher (80.1%, 1831/2287), as expected.

**Table 1:** Information of studies included in the Meta-analysis.

No	Author	Publishing date	Region	Race	Genotype	Treatment duration	Gender (% for male)	Age	SVR	Total
**Treatment-naïve patients**										
1	Ge et al [4]	Aug-09	USA	African	1	48W	62.8%	50.1±6.5^a^	45	191
2				Caucasian	1	48W	62.0%	47.5±7.2	488	871
3				Hispanic	1	48W	61.3%	45.3±9.2	38	75
4	Hayes et al [14]	Nov-10	Japan	Asian	1b	48W	56.5%	58(51–65)^c^	366	812
5	Kawaoka et al [15]	Sep-10	Japan	Asian	2	24W	N/A	N/A	99	130
6	Kurosaki et al [19]	Sep-10	Japan	Asian	1b	≥24W	50.4%	57.1±9.9	194	496
7	Mangia et al [8]	Jun-10	Italy	Caucasian	2/3	12/24W	57.8%	≥40:77.6%	201	268
8	Scherzer et al [20]	Jan-11	Austria	Caucasian	3	24W	63.9%	36.2±9.3	53	69
9	Yu et al [21]	Jan-11	Taiwan	Asian	2	24W	54.8%	52.8±11.1	429	482
10	Moghaddam et al [17]	Mar-11	Norway,Denmark	Caucasian	3	14–24W	59.1%	>40:39.5%	226	281
11	Fattovich et al [22]	Mar-11	Italy	Caucasian	1	48W	64.5%	46±11	65	121
12				Caucasian	2/3	24W	55.3%	46±11	144	159
13	Lin et al [16]	Mar-11	Taiwan	Asian	1	24W	64.4%	51.4±11.1	131	191
14	Bitetto et al [23]	Apr-11	Italy	Caucasian	1/4/5	48W	51.7%	47 (18–77)^c^	47	110
15				Caucasian	2/3	24W			87	101
16	Lindh et al [24]	Jun-11	Denmark, Finland, Norway and Sweden	Caucasian	2/3	12/24W	59.8%	42 (20–72)^c^	237	341
17	Mangia et al [25]	May-11	Italy	Caucasian	1	24/48/72W	57.7%	50.2	230	454
18	de Rueda et al [26]	Jun-11	Spain	Caucasian	1/4	48W	58.2%	>40:61.9%	198	373
19				Caucasian	2/3	48W			41	50
20	Ladero et al [27]	Jun-11	Spain	Caucasian	1	48W	62.7%	48 (20–74)^c^	51	110
21	Sarrazin et al [28]	Jul-11	Germany	Caucasian	1	24–72W	55.2%	43 (18–73)^c^	291	542
22	Suppiah et al [29]	Sep-11	Australia	Caucasian	1	48W	62.5%	SVR:40.9±10.8;N-SVR:45.7±9.3	389	848
23	Sandra et al [30]	Sep-11	Austria	Caucasian	1	48W	55.0%	46.4±12.2	51	113
24	De Nicola et al [31]	Sep-11	Italy	Caucasian	4	48W	83.6%	46 (27–63)^c^	50	103
25	Howell et al [32]	Oct-11	USA	African	1	48W	53.8%	49.0±7.0	45	173
26				Caucasian	1	48W	60.6%	47.2±8.5	101	188
27	Umemura et al [33]	Jul-11	Japan	Asian	1	48W	46.2%	58 (17–74)^c^	22	52
28	Scherzer et al [34]	Nov-11	Austria	Caucasian	1/4	24/48/72W	64.7%	44.4±10.8	182	328
**Patients with unsuccessful or unknown treatment history**										
29	Miyaaki et al [35]	Nov-11	Japan	Asian	1b	48W	55.2%	56.8±9.3	29	67
30	Lindh et al [36]	Nov-10	Sweden	Caucasian	1	24–72W	60.9%	45.4^d^	63	106
31	Hayashi et al [37]	Jun-11	Japan	Asian	1b	48W	52.5%	55.9±10.3	138	299
32	Halfon et al [38]	Oct-11	Israel	Caucasian	1	48W	70.7%	47±12	77	156
33				Caucasian	2/3	24W			31	42
34	Lyoo et al [39]	Sep-11	South Korea	Asian	1	48W	56.9%	52.4±9.7	42	65
35	Sakamoto et al [40]	Feb-11	Japan	Asian	2	24W	49.6%	64 (20–73)^b^	98	129
36	Sinn et al [41]	Mar-11	Korea	Asian	1	48W	53.4%	56.6±9.2	35	55
37				Asian	2	24W			52	63
38	O'Brien et al [42]	Jul-11	USA	Caucasian	1	48W	75.4%	49 (45–53)^b^	92	646
**HIV/HCV co-infected patients**										
39	Pineda et al [43]	Sep-10	Spain	Caucasian	1/4	48W	84.4%	42 (38–44)^c^	33	103
40				Caucasian	2/3	24/48W			44	51
41	Aparicio et al [44]	Oct-10	Spain	Caucasian	1/4	N/A	66.9%	SVR:48.4±0.7; Non-SVR:47.2±0.6	38	114
42				Caucasian	3				29	46
43	Rallón et al [45]	Apr-11	Spain	Caucasian	1/4	48/72W	75.0%	42 (38–45)^b^	57	135
44				Caucasian	2/3	24/48W			47	60
45	Labarga et al [46]	May-11	Spain	Caucasian	1/4	48W	82.3%	43^d^	12	47
46				Caucasian	2/3	48W			13	15

Age were described as

a mean ± standard deviation,

b median (interquartile range),

c median (range) and

d mean age.

N/A: not available.

### Treatment-naïve patients

Studies on the HCV G1/4 and the HCV G2/3 treatment-naïve patients were analyzed separately both for rs12979860 and rs8099917. The summarized SVR rate was 48.5% (2984/6151) for G1/4 group and 80.6% (1517/1881) for G2/3 group.

**Figure 2:**
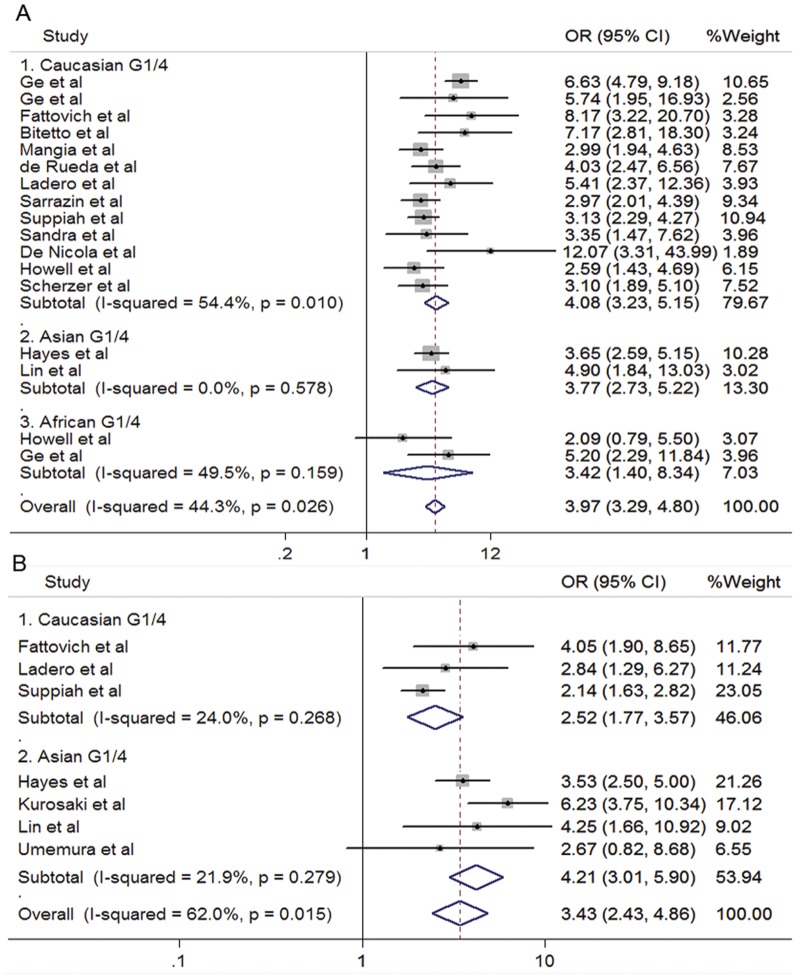
Forrest plots for association between polymorphisms and response to PegIFN/RBV in treatment-naïve HCV G1/4 patients. A. rs12979860 (CC vs CT/TT). B. rs8099917 (TT vs TG/GG).

**Figure 3:**
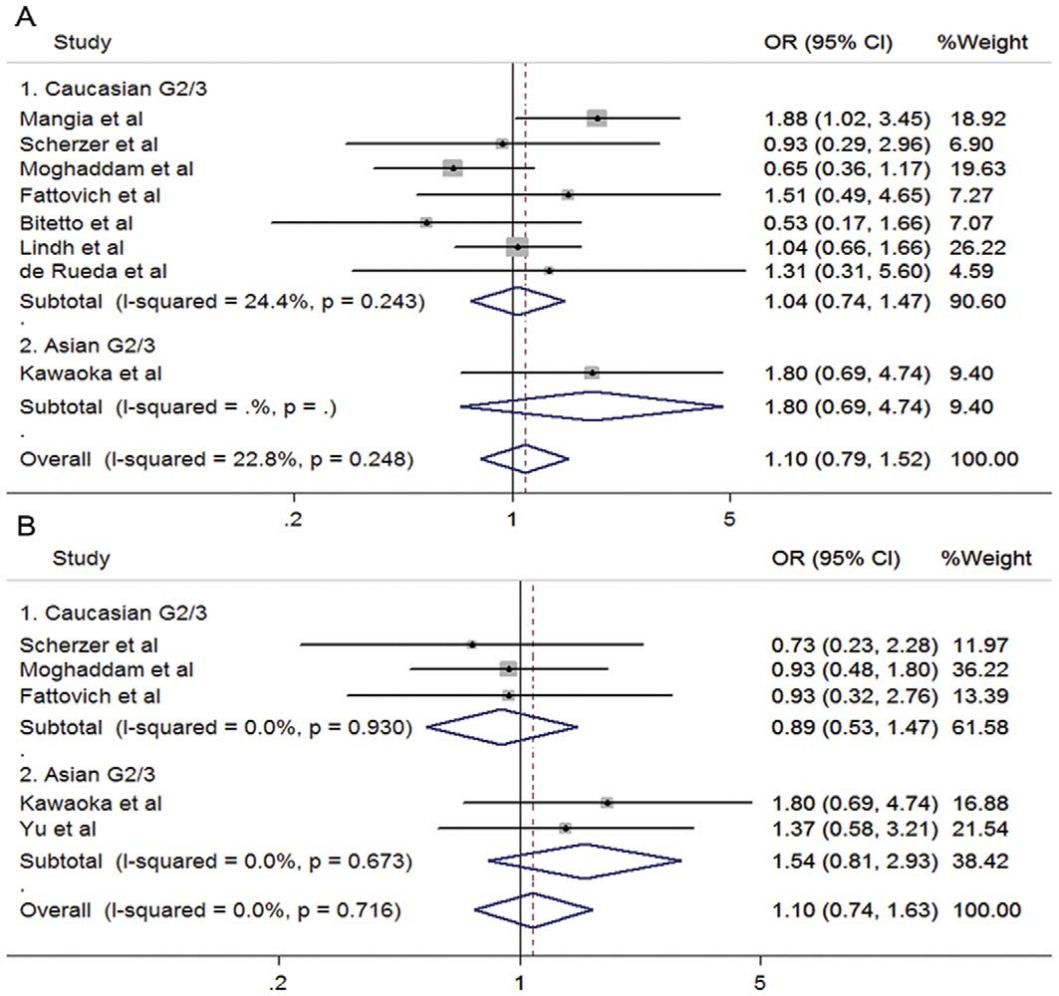
Forrest plots for association between polymorphisms and response to PegIFN/RBV in treatment-naïve HCV G2/3 patients. A. rs12979860 (CC vs CT/TT). B. rs8099917 (TT vs TG/GG).

Seventeen studies evaluated the association between response of combined therapy and SNP of rs12979860 in HCV G1/4 patients (Figure 2). When data from Caucasian were combined, the CC genotype showed a favorable effect compared to CT/TT (SVR: 73.1% vs 40.5%; OR 4.08, 95%CI 3.23–5.15; *P*<0.001). The favorable effects of CC genotype were also observed in Asian (SVR: 57.8% vs 24.8%; OR 3.77, 95%CI 2.73–5.22; *P*<0.001) and African patients (SVR: 48.0% vs 21.0%; OR 3.42, 95%CI 1.40–8.34; *P* = 0.007). The summarized OR from all studies indicated that rs12979860 CC genotype was associated with an increased possibility of SVR in the treatment-naïve patients with HCV genotype 1 or 4 infection, when they received the PegIFN/RBV treatment (OR 3.97, 95%CI 3.29–4.80, *P*<0.001; Figure 2). For patients of HCV G2/3, associations could not be observed either in Caucasian (SVR: 78.5% vs 77.5%; OR 1.04, 95%CI 0.74–1.47; *P* = 0.811) or Asian patients (SVR: 78.3% vs 66.7%; OR 1.80, 95%CI 0.69–4.74; *P* = 0.231). Neither was a significant association found in the pooled estimation (OR 1.10, 95%CI 0.79–1.52, *P* = 0.572; Figure 3).

We next grouped studies on Caucasian patients with complete SVR data for each rs12979860 genotype (Table 2). There was no Asian aggregation due to the limited number of studies. The SVR rates declined as the frequencies of C allele within HCV G1/4 or G2/3 group decreased. And the SVR rates were always higher in HCV G2/3 patients than in G1/4 patients for each rs12979860 genotype. For rs12979860 genotype distribution, the frequency of favorable CC genotype was higher in HCV G2/3 whereas the unfavorable CT or TT genotype was reversed (Table 2). The pooled estimations from these Caucasian studies were similar to those from all studies. The ORs were 4.09 (95% CI 2.98–5.62; *P*<0.001) for HCV G1/4 group and 1.03 (95% CI 0.70–1.51; *P* = 0.893) for HCV G2/3 group respectively.

**Table 2:** SVR rates and frequency for rs12979860 genotypes in Caucasian treatment-naïve patients with complete data.

	Reference	CC	CT	TT
**SVR**				
G1/4 (51.2%)	[4], [22], [23], [25], [27], [28], [29], [32]	72.7% (793/1090)	41.6% (684/1646)	34.4% (156/454)
G2/3 (77.8%)	[8], [17], [20], [22], [23], [24]	78.3% (403/515)	78.6% (453/576)	71.9% (92/128)
**Genotype frequency**				
G1/4	[4], [22], [23], [25], [27], [28], [29], [32]	34.2% (1090/3190)	51.6% (1646/3190)	14.2% (454/3190)
G2/3	[8], [17], [20], [22], [23], [24]	42.2% (515/1219)	47.3% (576/1219)	10.5% (128/1219)

Similar results were observed for rs8099917 TT genotype in chronic hepatitis C patients infected with HCV genotype 1 or 4. Compared to TG/GG genotypes, individuals bearing rs8099917 TT genotype were more likely to clear virus after PegIFN/RBV treatment both in Caucasian and Asian groups (Caucasian: 56.8% vs 35.8%, OR 2.52, 95%CI 1.77–3.57, *P*<0.001; Asian: 54.9% vs 21.3%, OR 4.21, 95%CI 3.01–5.90, *P*<0.001; Figure 2). The summarized OR for all studies was 3.43 with 95% CI from 2.43 to 4.86 (*P*<0.001, Figure 2). Again, no significant association was observed in patients infected with HCV genotype 2 or 3 (OR 1.10, 95%CI 0.74–1.63, *P* = 0.648; Figure 3).

### Patients with unsuccessful or unknown treatment history

**Figure 4:**
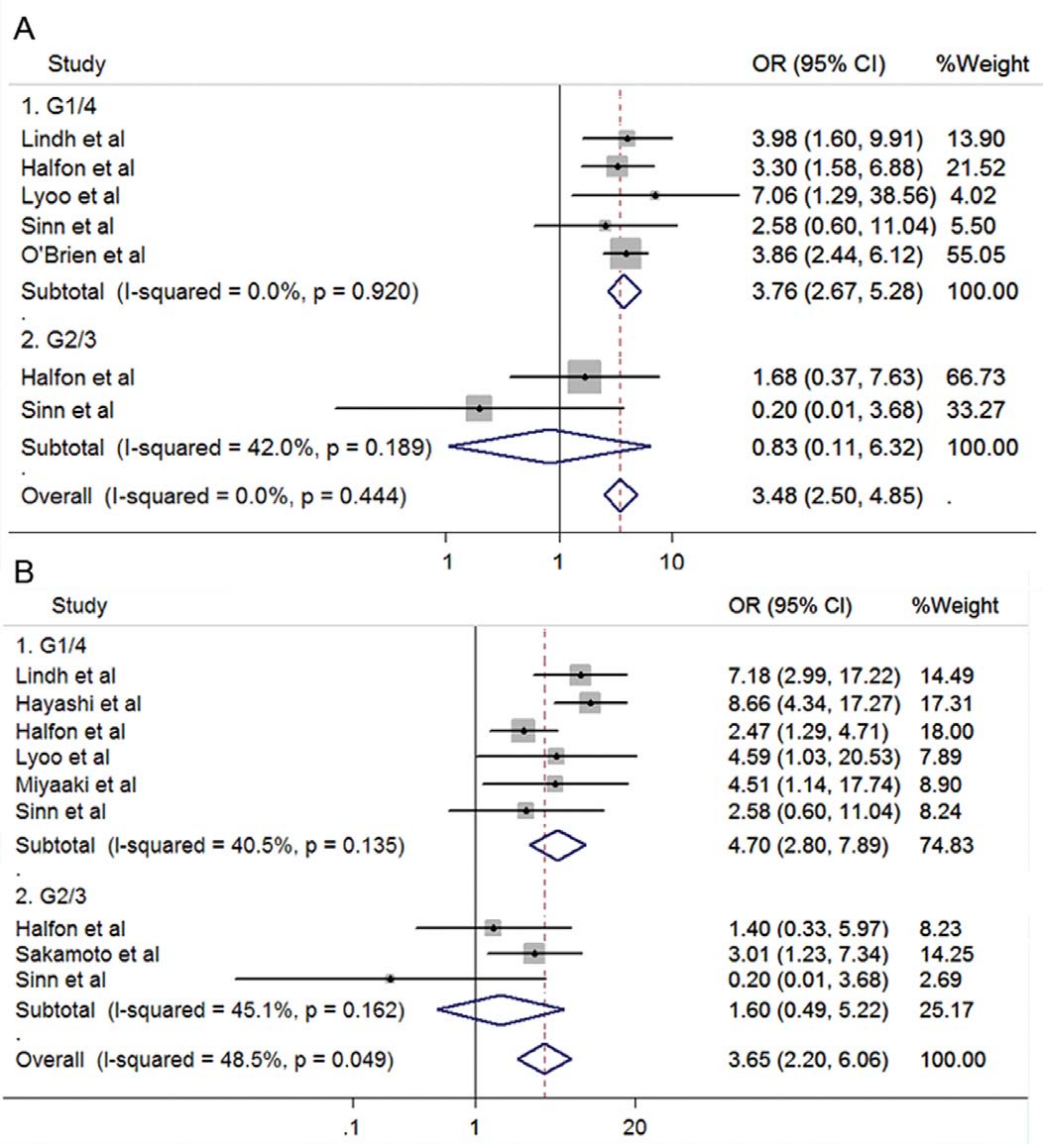
Forrest plots for association between polymorphisms and response to PegIFN/RBV in patients possibly with treatment history. A. rs12979860 (CC vs CT/TT). B. rs8099917 (TT vs TG/GG).

Studies were grouped together if they contained at least one patient failed to a previous interferon-based treatment or if no information of treatment history was given. This group was to show the impact of SNPs in the complex clinical practice since treatment response was reported to be different in patients with treatment history [2]. As expected, the summarized SVR rates were slightly lower than those in treatment-naïve patients, 34.1% (476/1394) in HCV G1/4 and 77.4% (181/234) in HCV G2/3 group. However, associations between SNPs and treatment effects were similar to that in the treatment-naïve group. The positive associations were observed in HCV G1/4 group carrying rs12979860 CC (OR 3.76, 95%CI 2.67–5.28, *P*<0.001) or rs8099917 TT genotype (OR 4.70, 95%CI 2.80–7.89, *P*<0.001) respectively, whereas no significant associations was found in HCV G2/3 group (Figure 4).

### HCV/HIV co-infected patients

**Figure 5:**
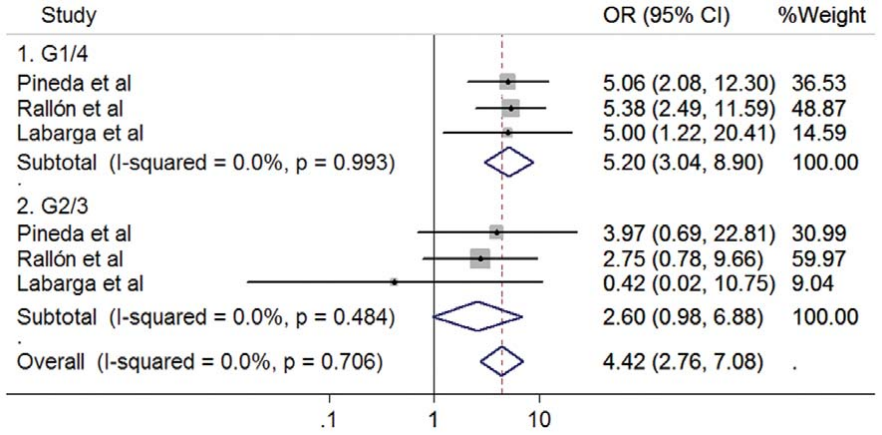
Forrest plot for association between rs12979860 polymorphisms and response to PegIFN/RBV in HIV co-infected patients.

Studies conducted in HCV/HIV co-infected patients were classified into one group, without considering the treatment history due to the similar results observed in HCV mono-infected patients with and without treatment history and the limited number of studies. The pooled results indicated similar associations as those in HCV mono-infected patients. The rs12979860 CC genotype was associated with a better treatment response in HCV G1/4 patients receiving PegIFN/RBV therapy (OR 5.20, 95%CI 3.04–8.90, *P*<0.001; Figure 5), but not in HCV G2/3 patients (OR 2.60, 95%CI 0.98–6.88, *P* = 0.054). However, only one study was on rs8099917, and this study showed that re8099917 TT genotype favored the positive association in patients with HCV genotype 1 or 4, but not in genotype 2 or 3 [44].

### Publication bias

No obvious publication bias was observed in studies using Egger's test and Peters' test respectively, as shown in table 3.

**Table 3:** Results for publication bias test using Egger's test and Peters' test.

Group	*P* for Egger	*P* for Peters
**Rs12979860**		
HCV G1/4 and treatment- naïve patient	0.310	0.286
HCV G2/3 and treatment- naïve patient	0.889	0.946
Patients with unsuccessful or unknown treatment history	0.167	0.291
HIV/HCV co-infected patient	0.043	0.342
**Rs8099917**		
HCV G1/4 and treatment- naïve patient	0.331	0.879
HCV G2/3 and treatment- naïve patient	0.960	0.532
Patients with unsuccessful or unknown treatment history	0.189	0.143

## Discussion

In the present study, we collected and summarized studies on associations between SNPs near IL28B gene (rs12979860 and rs8099917) and the treatment effect of PegIFN/RBV in chronic HCV patients. Roughly, a 3-fold significant increase of possibility to clear virus (SVR) was observed for rs12979860 CC genotype or rs8099917 TT genotype in patients infected with HCV genotype 1 or 4, irrespective of race, treatment history or the status of HIV co-infection. However, no relationship was found in patients infected with HCV genotype 2 or 3. A possible interpretation is that the effect of SNPs is attenuated by a better treatment response in patients with genotype 2 or 3 HCV as several studies have reported that rs12979860 CC or rs8099917 TT genotype was associated with faster early viral elimination in patients with genotype 2 or 3 HCV [17], [20], [21], [24] and the rate of SVR in treatment-naïve subjects infected with genotype 2 or 3 is 80.6%, much higher than 48.5% in patients with HCV genotype 1 or 4.

Rs12979860, located about 3 kb upstream to the IL28B coding region, resides in the putative promoter or the regulatory region of IL28B gene and may affect the gene expression as predicted by a bioinformatics tool, FastSNP (http://fastsnp.ibms.sinica.edu.tw/pages/input_SNPListAnalysis.jsp). The C allele has been reported to be linked to higher serum levels of IL28B, IL28A and IL29 levels, which are all involved in the induction of expression of IFN-responsive genes [47]. The CC genotype has also been reported to be associated with lower levels of intrahepatic interferon-stimulated gene (ISG) expression, which is linked to better IFN-based treatment response [48]. However, the exact mechanism underlying effects of the SNP on treatment response still remains to be clarified. Nonetheless, several facts are noteworthy. Frequencies of favorable CC genotype of rs12979860 vary in different races, but in the same direction as SVR rates to PegIFN/RBV treatment [49], [50]. And the frequencies of CC genotype increase from the lowest in patients failed to treatment, to intermediate in patients with treatment-induced clearance, and then to the highest in patients with spontaneous clearance [50], [51]. In our analysis, the SVR rate declines with the reduction of C allele (72.7% for CC, 41.6% for CT, 34.4% for TT, Table 2) when data from the Caucasian HCV G1/4 treatment-naïve patients were combined. Rs8099917 has been reported to be in strong linkage disequilibrium with rs12979860 [4], [14]–[18]. Similar associations with HCV treatment are observed and the predictive value could not be increased when determined simultaneously [38]. Therefore, rs8099917 may share similar mechanism as rs12979860 on the association with treatment response although the mechanism is still unclear and determination of the genotype of one SNP may be enough in clinical practice.

Limitations and cautions should be taken into account before making conclusions. Firstly, selection of patients could cause a bias. Several studies analyzed patients with compliance >80% in all patients [16], [17], [21], [24], [38] or >80% only in non-SVR patients [4], [19], [22]. Non-responders are more likely to discontinue therapy prematurely [3] and the frequencies of unfavorable genotypes tend to be higher in non-responders [50], [51]. Therefore, selection of patients according to the compliance may result in an underestimation of SNP effect on treatment response. Secondly, therapeutic effect could be influenced by multiple variables known or unknown. For example, baseline HCV RNA level has been recognized to be reversely correlated with treatment effect [4]. In this analysis, we could not adjust this factor because different studies applied different criteria to define high and low levels of HCV RNA [4], [15], [16], [23]. Therefore, the unified design should be considered in future studies to calculate the net contribution of IL28B to treatment response. Thirdly, a group of patients with unsuccessful or unknown treatment history in our review was meant to represent the actual complex clinical setting. The interpretation of results from this group should be cautious since the portions of patients with treatment history were different among different studies. However, the study carried out in patients all failed to previous treatment also supported the association concluded in our analysis [42], indicating the predictable values of SNP near the IL28B gene. Finally, several studies were excluded from our analysis due to no sufficient data to calculate the ORs stratified by races and HCV genotypes according to our criteria [50]–[54]. However, the same conclusion would be derived if these studies were included, only with the values of ORs slightly altered, since these studies also favor the results we get.

In summary, our findings suggest that SNPs of IL28B are useful baseline predictors for virological response in patients infected with genotype 1 or 4 HCV when treated with PegIFN/RBV, but not in patients with genotype 2 or 3 HCV. Therefore, determination of IL28B genotype may be necessary only in HCV genotype 1 or 4 patients. For those patients infected with difficult-to-treat genotype 1 or 4 HCV and also bearing rs12979860 non-CC genotype or rs8099917 non-TT genotype, it is urgent to develop more effective therapy strategies.

## Supporting Information

File S1 **Form for paper assessment and data collection.**
(DOC)
