# Supplementary material for: Test of IL28B Polymorphisms in Chronic Hepatitis C Patients Treated with PegIFN and Ribavirin Depends on HCV Genotypes: Results from a Meta-Analysis
Source: PLoS One. 2012 Sep 21;7(9):e45698. doi: 10.1371/journal.pone.0045698 (PMC3448689; doi:10.1371/journal.pone.0045698)
Supplement: File S1 — Form for paper assessment and data collection. (DOC) [file pone.0045698.s001.doc]

**Form for paper assessment and data collection**

No. of Article:______ Reviewer :________

1. First Author: ____________

2. Date of publishing: _____-___-___

3. Region of the patients: ____________ or □Not mentioned

4. Ethnicity of the patients: □Caucasian □Asian □Hispanic

□African □Not mentioned

5. Chronic HCV patients：□Yes □No □Not mentioned

6. HCV genotype of patients: __________ or □Not mentioned

7. Baseline HCV RNA level was detected: □Yes □No

8. Treatment was combination of PegIFN/RBV only: □Yes □No

9. SNP sites studied: □rs12979860 □rs8099917

□both of above □others

10. Clearly defined outcome as SVR: □Yes □No

11. Availability of Data of SVR for SNP genotypes: □Yes □No

12. Overlapping with other studies: □Yes □No

If yes, the No. of the articles: ______________

**Eligibility of the study: □Yes □No □Further consideration needed**

If the paper was not excluded after assessing above, more detailed information for this paper:

13. Source of patients: □Clinical trial □Clinical routine

□Not mentioned

14. Patients of treatment-naïve: □Yes □Part or all had treatment history

□Not mentioned

15. Co-infection with HIV: □Yes □No

16. Method of detecting SNPs:______________

17. Number of independent studies of this paper: ________

18. Other information worth recording: __________________________

**19. Final decision on this paper: □Included □Excluded**

If included, the group classed into: □1. Treatment-naive

□2. Real patients

□3. HIV co-infected

If excluded, the reason: __________________________________

20. Data for rs12979860

| Ethnicity | HCV genotype | Duration of treatment | Gender | age | SVR | | | Non- SVR | | |
| --- | --- | --- | --- | --- | --- | --- | --- | --- | --- | --- |
|  |  |  |  |  | CC | CT | CT | CC | CT | TT |
|  |  |  |  |  |  |  |  |  |  |  |
|  |  |  |  |  |  |  |  |  |  |  |
|  |  |  |  |  |  |  |  |  |  |  |
|  |  |  |  |  |  |  |  |  |  |  |
|  |  |  |  |  |  |  |  |  |  |  |

21. Data for rs8099917

| Ethnicity | HCV genotype | Duration of treatment | Gender | age | SVR | | | Non- SVR | | |
| --- | --- | --- | --- | --- | --- | --- | --- | --- | --- | --- |
|  |  |  |  |  | TT | TG | GG | TT | TG | GG |
|  |  |  |  |  |  |  |  |  |  |  |
|  |  |  |  |  |  |  |  |  |  |  |
|  |  |  |  |  |  |  |  |  |  |  |
|  |  |  |  |  |  |  |  |  |  |  |
|  |  |  |  |  |  |  |  |  |  |  |
